# Supplementary figures and images for: Uncovering hidden specific diversity of Andean glassfrogs of the Centrolene buckleyi species complex (Anura: Centrolenidae)
Source: PeerJ. 2018 Oct 31;6:e5856. doi: 10.7717/peerj.5856 (PMC6215445; doi:10.7717/peerj.5856)

GMYC  
single threshold  
Results

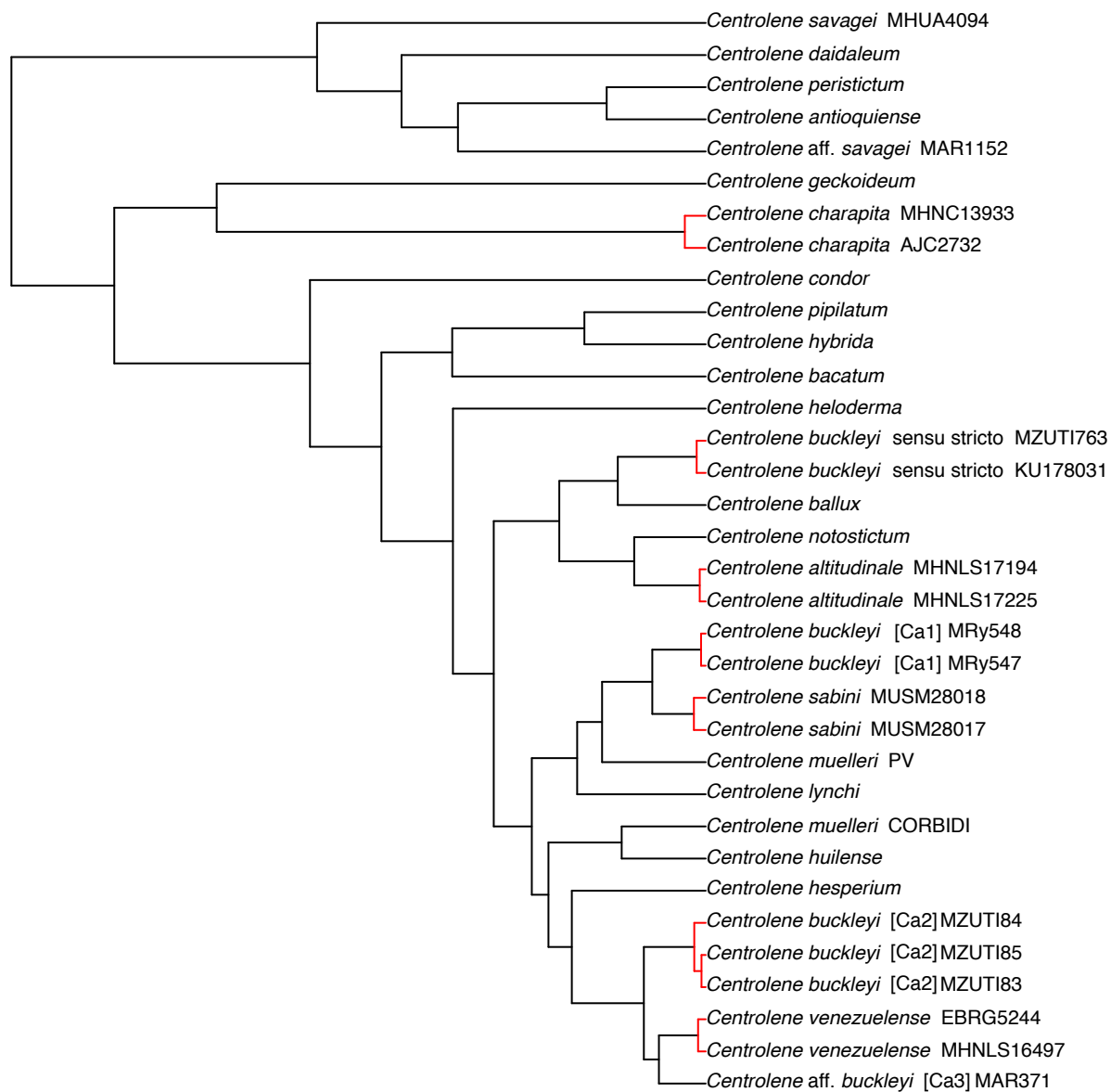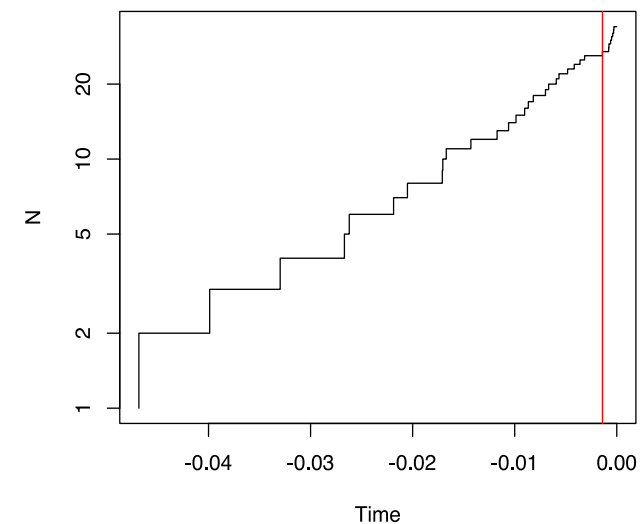

Supplement: Supplemental Information 1 — Each terminal in the tree represents a molecular entity delimited. Red clades represent a single molecular entity delimited with this method. [file peerj-06-5856-s001.pdf]

GMYC  
multiple threshold  
Results

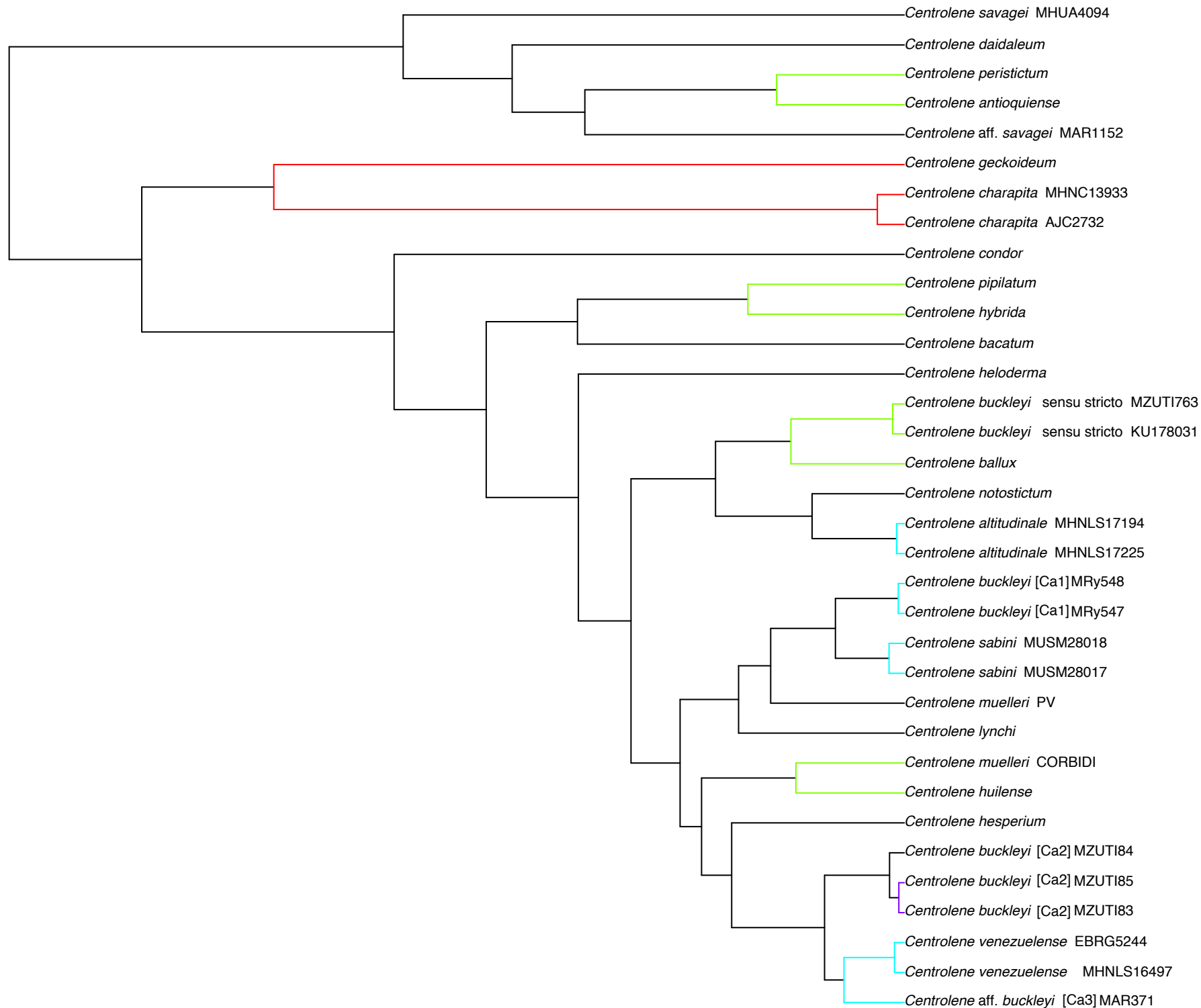

Supplement: Supplemental Information 2 — Each terminal in the tree represents a molecular entity delimited. Colored clades represent a single molecular entity delimited with this method. [file peerj-06-5856-s002.pdf]

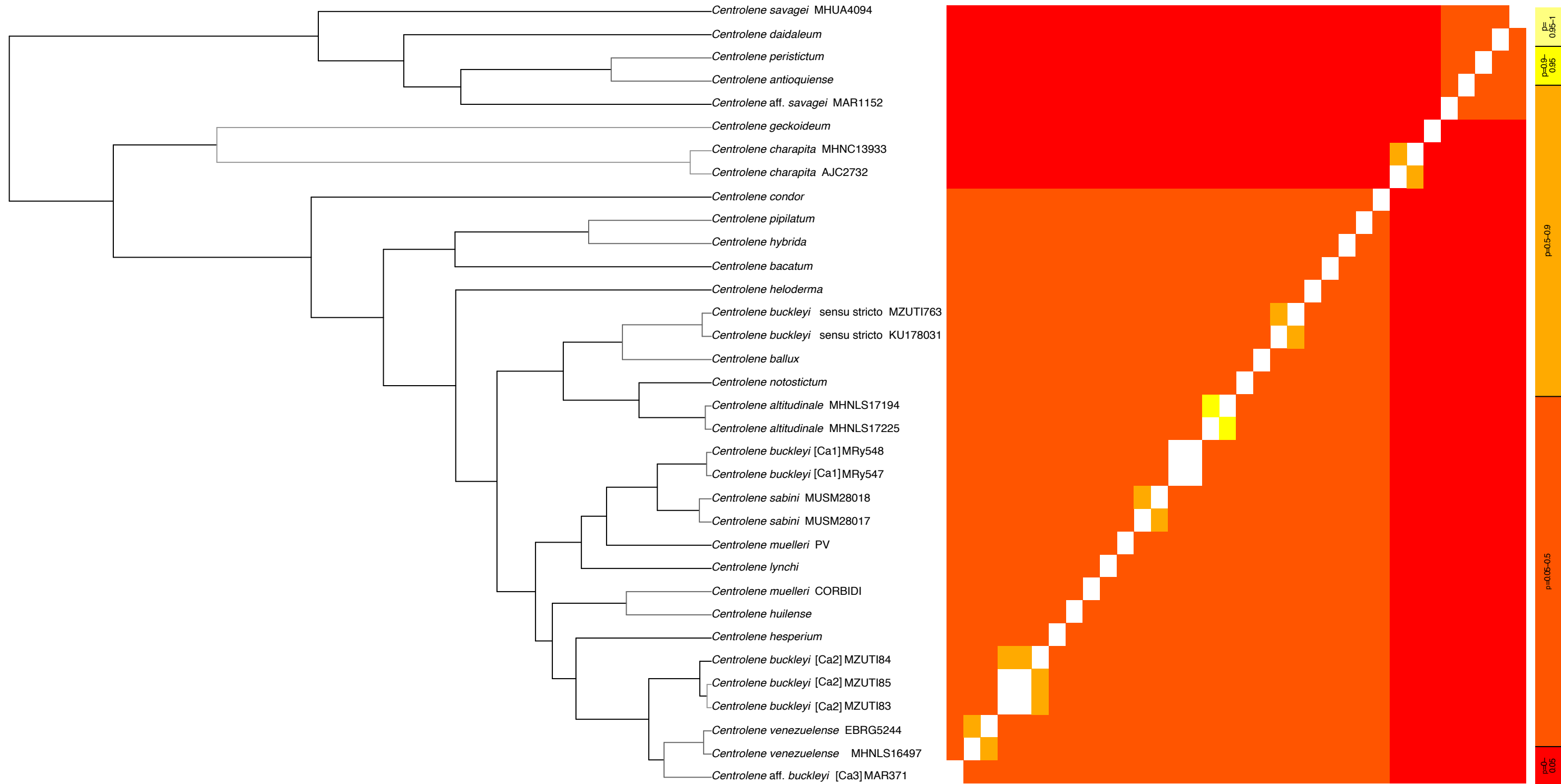

Supplement: Supplemental Information 3 — The Klee plot is a sequence-by-sequence matrix where cells are coloured by pairwise posterior probabilities of conspecificity between sequence pairs. [file peerj-06-5856-s003.pdf]

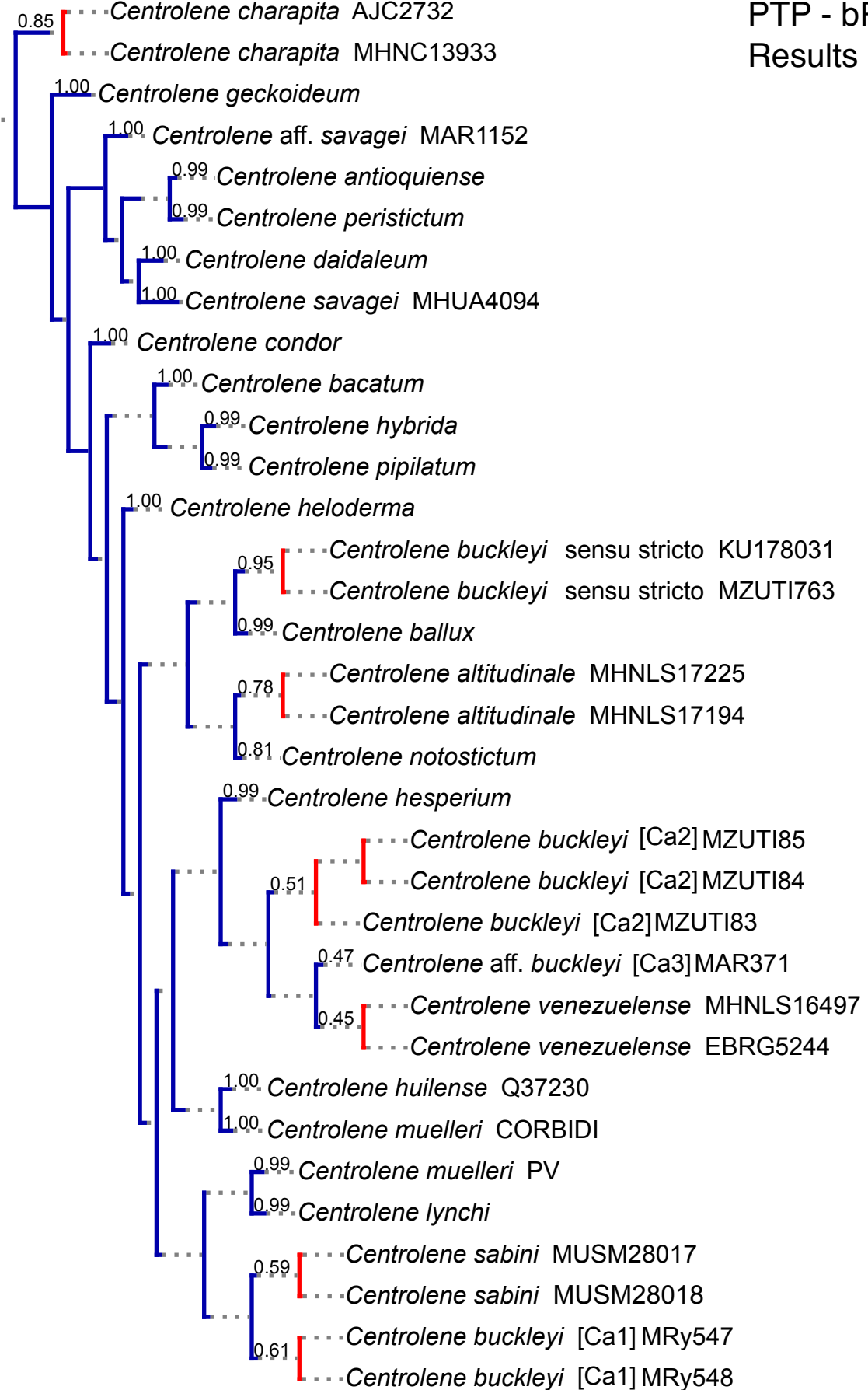

0.10

Supplement: Supplemental Information 4 — Each terminal in the tree represents a molecular entity delimited. Red clades represent a single molecular entity delimited with this method. [file peerj-06-5856-s004.pdf]

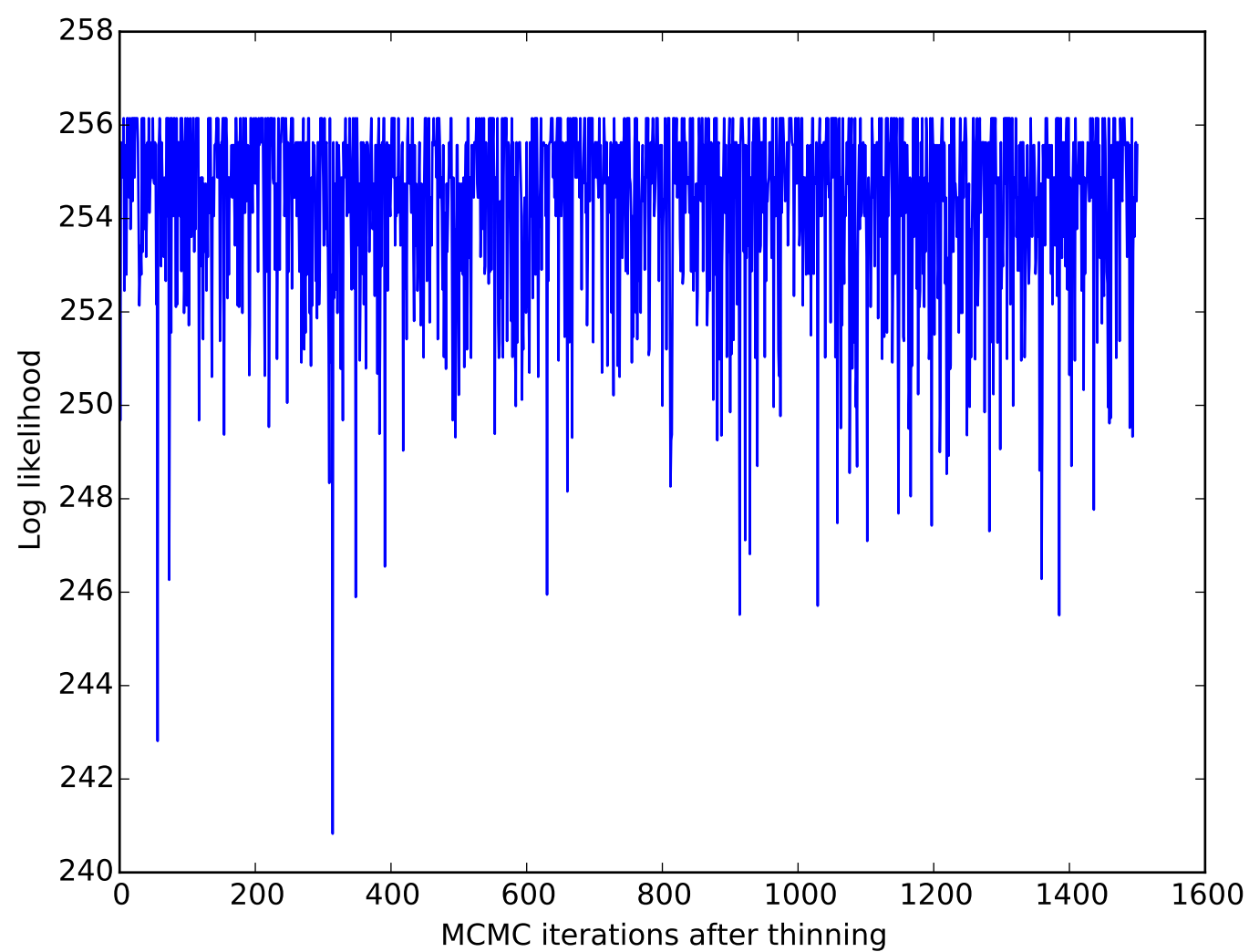

Supplement: Supplemental Information 5 [file peerj-06-5856-s005.pdf]
