# Supplementary material for: Uncovering hidden specific diversity of Andean glassfrogs of the Centrolene buckleyi species complex (Anura: Centrolenidae)
Source: PeerJ. 2018 Oct 31;6:e5856. doi: 10.7717/peerj.5856 (PMC6215445; doi:10.7717/peerj.5856)
Supplement: Supplemental Information 6 [file peerj-06-5856-s006.docx]

Table S1. Voucher numbers and GenBank accession numbers of all specimens included in the study

| Species | ID /voucher number | 12S | 16S | References |
| --- | --- | --- | --- | --- |
| *Centrolene* aff. *buckleyi*  [Ca3] | MAR 371 | EU663339 | EU662980 | Guayasamin et al. 2008 |
| *Centrolene altitudinale* | MHNLS 17225 | EU663334 | EU662975 | Guayasamin et al. 2008 |
| *Centrolene altitudinale* | MHNLS 17194 | EU663333 | EU662974 | Guayasamin et al. 2008 |
| *Centrolene antioquiense* | NRPS 014 | EU663336 | EU662977 | Guayasamin et al. 2008 |
| *Centrolene bacatum* | QCAZ 22728 | EU663337 | EU662978 | Guayasamin et al. 2008 |
| *Centrolene ballux* | QCAZ 40196. QCAZ 40182 | KF639754 | JX126954 | Castroviejo-Fisher et al. 2014 |
| *Centrolene buckleyi* | KU 178031 | EU663338 | EU662979 | Guayasamin et al. 2008 |
| *Centrolene buckleyi* | MZUTI 763 | MH844843 | MH844849 | Present work |
| *Centrolene charapita* | AJC 2732 | KF639760 | KF534358 | Castroviejo-Fisher et al. 2014 |
| *Centrolene charapita* | MHNC 13933 | KM068247 | KM068256 | Twomey et al. 2014 |
| *Centrolene condor* | QCAZ 44896 | KF639755 | JX126955 | Catenazzi et al. 2012; Castroviejo-Fisher et al. 2014 |
| *Centrolene daidaleum* | MHUA 3271 | EU663366 | EU663007 | Guayasamin et al. 2008 |
| *Centrolene geckoideum* | KU 178015 | EU663341 | EU662982 | Guayasamin et al. 2008 |
| *Centrolene heloderma* | QCAZ 40200 | KF639757 | JX126956 | Catenazzi et al. 2012; Castroviejo-Fisher et al. 2014 |
| *Centrolene hesperium* | MHNSM 25802 | EU663345 | EU662986 | Guayasamin et al. 2008 |
| *Centrolene huilense* | QCAZ 37230 |  | JX126959 | Catenazzi et al. 2012 |
| *Centrolene hybrida* | MAR 347 | EU663346 | EU662987 | Guayasamin et al. 2008 |
| *Centrolene lynchi* | QCAZ 40191. QCAZ 40192 | KF639758 | JX126957 | Catenazzi et al. 2012; Castroviejo-Fisher et al. 2014 |
| *Centrolene muelleri* | PV |  | JX126958 | Catenazzi et al. 2012 |
| *Centrolene muelleri* | CORBIDI 14667 | KF639759 | KM068267 | Castroviejo-Fisher et al. 2014; Twomey et al. 2014 |
| *Centrolene notostictum* | MAR 510 | EU663351 | EU662992 | Guayasamin et al. 2008 |
| *Centrolene peristictum* | QCAZ 22312 | EU663352 | EU662993 | Guayasamin et al. 2008 |
| *Centrolene pipilatum* | KU 178154 | EU663353 | EU662994 | Guayasamin et al. 2008 |
| *Centrolene sabini* | MUSM 28017 |  | JX126961 | Catenazzi et al. 2012 |
| *Centrolene sabini* | MUSM 28018 |  | JX126960 | Catenazzi et al. 2012 |
| *Centrolene* aff. *savagei* | MAR1152 | KM068295 | KM068295 | Twomey et al. 2014 |
| *Centrolene savagei* | MHUA 4094 | EU663380 | EU663020 | Guayasamin et al. 2008 |
| *Centrolene buckleyi* [Ca1] | MRy 547 | MH844838 | MH844844 | Present work |
| *Centrolene buckleyi* [Ca1] | MRy 548 | MH844839 | MH844845 | Present work |
| *Centrolene buckleyi* [Ca2] | MZUTI 83 | MH844840 | MH844846 | Present work |
| *Centrolene buckleyi* [Ca2] | MZUTI 84 | MH844841 | MH844847 | Present work |
| *Centrolene buckleyi* [Ca2] | MZUTI 85 | MH844842 | MH844848 | Present work |
| *Centrolene venezuelense* | EBRG5244 | EU663359 | EU663000 | Guayasamin et al. 2008 |
| *Centrolene venezuelense* | MHNLS 16497 | EU663360 | EU663001 | Guayasamin et al. 2008 |
| *Nymphargus bejaranoi* | CBG 1488 | EU663422 | EU663059 | Guayasamin et al. 2008 |
| *Nymphargus posadae* | QCAZ 26023. QCAZ 25090 | KF639770 | KF534367 | Castroviejo-Fisher et al. 2014 |
| *Nymphargus ocellatus* | GCI363 | KF639769 | KF534366 | Castroviejo-Fisher et al. 2014 |
| *Nymphargus pluvialis* | KU 173224 | EU663428 | EU663065 | Guayasamin et al. 2008 |
| *Nymphargus rosada* | MHUA 4308 | EU663429 | EU663066 | Guayasamin et al. 2008 |
| *Nymphargus anomalus* | QCAZ 41312. QCAZ 45703 | KF639766 | KF534364 | Castroviejo-Fisher et al. 2014 |
| *Nymphargus siren* | KU 179171 | EU663430 | EU663067 | Guayasamin et al. 2008 |
| *Nymphargus megacheirus* | KU 143272 | EU663427 | EU663063 | Guayasamin et al. 2008 |
| *Nymphargus wileyi* | QCAZ 27435 | EU663431 | EU663068 | Guayasamin et al. 2008 |
| *Nymphargus lasgralarias* | QCAZ 11689 | KF208512 | KF208513 | Hutter et al. 2013 |
| *Nymphargus griffithsi* | QCAZ 29525 | KF208515 | KF208516 | Hutter et al. 2013 |
| *Nymphargus mixomaculatus* | MTD 45200 | KF639768 | EU663064 | Guayasamin et al. 2008 |
| *Nymphargus cochranae* | QCAZ 31113 | EU663425 | EU663061 | Guayasamin et al. 2008 |
