# Supplementary material for: Uncovering hidden specific diversity of Andean glassfrogs of the Centrolene buckleyi species complex (Anura: Centrolenidae)
Source: PeerJ. 2018 Oct 31;6:e5856. doi: 10.7717/peerj.5856 (PMC6215445; doi:10.7717/peerj.5856)
Supplement: Supplemental Information 7 [file peerj-06-5856-s007.docx]

|  |  | 1 | 2 | 3 | 4 | 5 | 6 | 7 | 8 | 9 | 10 | 11 | 12 | 13 | 14 | 15 | 16 | 17 | 18 | 19 | 20 | 21 | 22 | 23 | 24 |
| --- | --- | --- | --- | --- | --- | --- | --- | --- | --- | --- | --- | --- | --- | --- | --- | --- | --- | --- | --- | --- | --- | --- | --- | --- | --- |
| 1 | *C.* aff. *buckleyi* [Ca3] |  |  |  |  |  |  |  |  |  |  |  |  |  |  |  |  |  |  |  |  |  |  |  |  |
| 2 | *C. altitudinale* | 1.7 |  |  |  |  |  |  |  |  |  |  |  |  |  |  |  |  |  |  |  |  |  |  |  |
| 3 | *C. antioquiense* | 6.3 | 6.1 |  |  |  |  |  |  |  |  |  |  |  |  |  |  |  |  |  |  |  |  |  |  |
| 4 | *C. bacatum* | 2.6 | 3.3 | 6.7 |  |  |  |  |  |  |  |  |  |  |  |  |  |  |  |  |  |  |  |  |  |
| 5 | *C. ballux* | 2.0 | 1.4 | 5.7 | 3.3 |  |  |  |  |  |  |  |  |  |  |  |  |  |  |  |  |  |  |  |  |
| 6 | *C. buckleyi* sensu stricto | 1.5 | 1.5 | 5.9 | 3.3 | 0.6 |  |  |  |  |  |  |  |  |  |  |  |  |  |  |  |  |  |  |  |
| 7 | *C. buckleyi* [Ca1] | 1.3 | 2.0 | 7.2 | 2.8 | 2.1 | 1.6 |  |  |  |  |  |  |  |  |  |  |  |  |  |  |  |  |  |  |
| 8 | *C. buckleyi* [Ca2] | 0.9 | 1.9 | 6.3 | 3.3 | 1.9 | 1.4 | 1.8 |  |  |  |  |  |  |  |  |  |  |  |  |  |  |  |  |  |
| 9 | *C. charapita* | 7.4 | 8.0 | 8.9 | 8.2 | 8.6 | 8.4 | 8.0 | 8.6 |  |  |  |  |  |  |  |  |  |  |  |  |  |  |  |  |
| 10 | *C. condor* | 3.1 | 2.9 | 6.2 | 2.9 | 3.1 | 2.9 | 3.3 | 3.9 | 8.8 |  |  |  |  |  |  |  |  |  |  |  |  |  |  |  |
| 11 | *C. daidaleum* | 6.7 | 5.9 | 4.1 | 6.3 | 6.2 | 6.1 | 7.0 | 6.2 | 10.1 | 6.0 |  |  |  |  |  |  |  |  |  |  |  |  |  |  |
| 12 | *C. geckoideum* | 5.4 | 4.9 | 5.8 | 5.2 | 5.7 | 5.1 | 5.9 | 5.7 | 9.4 | 4.7 | 6.3 |  |  |  |  |  |  |  |  |  |  |  |  |  |
| 13 | *C. heloderma* | 1.4 | 1.2 | 6.6 | 2.3 | 1.8 | 1.6 | 1.6 | 1.8 | 7.0 | 2.5 | 6.4 | 5.1 |  |  |  |  |  |  |  |  |  |  |  |  |
| 14 | *C. hesperium* | 1.5 | 2.0 | 6.7 | 3.3 | 2.7 | 2.2 | 2.0 | 1.6 | 8.2 | 3.7 | 6.4 | 5.5 | 2.0 |  |  |  |  |  |  |  |  |  |  |  |
| 15 | *C. huilense* | 2.5 | 2.9 | 7.4 | 3.5 | 3.1 | 2.9 | 2.7 | 2.9 | 8.2 | 4.1 | 6.8 | 6.7 | 2.3 | 2.9 |  |  |  |  |  |  |  |  |  |  |
| 16 | *C. hybrida* | 3.3 | 3.0 | 6.7 | 3.5 | 2.7 | 2.5 | 3.3 | 2.7 | 8.0 | 4.7 | 6.6 | 6.3 | 2.9 | 3.3 | 3.5 |  |  |  |  |  |  |  |  |  |
| 17 | *C. lynchi* | 1.6 | 2.1 | 7.2 | 2.5 | 2.3 | 1.8 | 1.0 | 2.0 | 8.2 | 3.5 | 6.6 | 5.7 | 1.8 | 2.2 | 2.3 | 3.5 |  |  |  |  |  |  |  |  |
| 18 | *C. muelleri* | 2.6 | 3.5 | 7.8 | 4.1 | 3.7 | 3.1 | 2.9 | 3.1 | 8.2 | 4.7 | 7.4 | 6.7 | 2.9 | 3.1 | 0.6 | 4.1 | 2.5 |  |  |  |  |  |  |  |
| 19 | *C. notostictum* | 2.1 | 0.7 | 7.8 | 3.6 | 1.7 | 1.5 | 2.4 | 2.7 | 9.5 | 3.6 | 8.0 | 5.4 | 1.5 | 2.9 | 3.6 | 3.6 | 2.7 | 4.4 |  |  |  |  |  |  |
| 20 | *C. peristictum* | 7.2 | 7.2 | 2.4 | 7.2 | 6.8 | 7.0 | 8.0 | 6.8 | 10.3 | 7.6 | 4.3 | 7.9 | 7.4 | 7.4 | 8.0 | 7.2 | 7.8 | 8.6 | 9.5 |  |  |  |  |  |
| 21 | *C. pipilatum* | 2.8 | 3.5 | 6.9 | 2.6 | 3.1 | 2.9 | 3.3 | 2.7 | 8.4 | 3.9 | 6.8 | 6.3 | 2.9 | 3.3 | 3.7 | 1.2 | 3.5 | 4.3 | 4.1 | 7.4 |  |  |  |  |
| 22 | *C. sabini* | 1.0 | 1.6 | 7.0 | 2.7 | 1.8 | 1.2 | 0.4 | 1.4 | 8.0 | 3.3 | 6.8 | 5.5 | 1.2 | 2.0 | 2.7 | 2.9 | 1.0 | 2.9 | 1.9 | 7.8 | 2.9 |  |  |  |
| 23 | *C. savagei* | 7.4 | 6.7 | 4.8 | 7.1 | 7.0 | 7.2 | 8.0 | 7.0 | 9.8 | 7.2 | 4.1 | 6.9 | 7.2 | 6.7 | 7.2 | 7.2 | 7.4 | 7.8 | 9.0 | 5.1 | 7.4 | 8.0 |  |  |
| 24 | *C. venezuelense* | 0.4 | 1.5 | 6.7 | 3.1 | 2.0 | 1.3 | 1.3 | 0.7 | 8.4 | 3.8 | 7.3 | 5.6 | 1.3 | 1.3 | 2.7 | 3.3 | 1.6 | 2.9 | 1.9 | 7.8 | 3.3 | 0.9 | 8.2 |  |
